# Defining Cystoid Macular Degeneration in Diabetic Macular Edema: An OCT-Based Single-center Study

**DOI:** 10.4274/tjo.galenos.2019.22687

**Published:** 2019-12-31

**Authors:** Gökçen Yalçın, Şengül Özdek, Fatma Nur Baran Aksakal

**Affiliations:** 1 Gazi University Faculty of Medicine, Department of Ophthalmology, Ankara, Turkey; 2 Gazi University Faculty of Medicine, Department of Public Health, Ankara, Turkey

**Keywords:** Diabetic macular edema, diabetic retinopathy, cystoid macular degeneration, chronic macular edema, optical coherence tomography

## Abstract

**Objectives:**

To describe cystoid macular degeneration (CMD), which has no clear definition in diabetic macular edema (DME), and examine its features in optical coherence tomography (OCT) and fundus fluorescein angiography (FFA).

**Materials and Methods:**

This study was conducted using OCT images of patients who were followed in Gazi University between November 2011 and March 2015. A total of 259 eyes (187 patients) found to have cystic changes on OCT were included. Macular ischemia, peripheral ischemia, and type of edema were identified on FFA. Vitreomacular interface abnormalities, foveal contour integrity, internal reflectivity of the cysts, and outer retinal layer defects were analyzed from OCT images. The horizontal and vertical diameters of the largest cyst within 1000 μm of the foveal center were measured for the definition of CMD. Cut-offs for these values were determined by receiver operating characteristic curve analysis. Cystoid macular edema (CME) and CMD groups were created and their characteristics were analyzed.

**Results:**

The horizontal and vertical diameters of the largest cyst were moderately positively correlated with visual acuity (rs=0.349, r=0.419, respectively). Eyes with horizontal diameter of the largest cyst ≥450 μm were classified as CMD; in this group, sensitivity in the prediction of visual acuity ≤20/60 was 58%. Eyes with horizontal diameter of the largest cyst <450 μm were classified as CME; in this group, specificity in the prediction of visual acuity >20/60 was 73%. For the threshold of 300 μm determined for vertical diameter of the largest cyst, sensitivity was 62% and specificity was 69%. The CME and CMD groups were formed according to these cut-off values. Compared to the CME group, the CMD group had greater central subfield thickness and higher prevalence of outer retinal damage, severe disruption of foveal contour, macular ischemia, and diffuse/mixed type edema.

**Conclusion:**

In eyes with DME, CMD can be defined as the largest cyst within 1000 μm of the foveal center having a horizontal diameter of ≥450 μm and vertical diameter ≥300 μm, especially if associated with macular ischemia, outer retinal damage, loss of foveal contour, and diffuse/mixed type edema.

## Introduction

Diabetic macular edema (DME) is the most common cause of visual deterioration in diabetic retinopathy (DR).^1^ The 25-year cumulative prevalence of DME in Type 1 DM was stated as 29% in a report of the Wisconsin Epidemiologic Study of DR.^2^ There are many definitions and classifications of types of DME, such as focal/diffuse edema, cystoid macular edema (CME), serous macular detachment, ischemic and tractional diabetic maculopathy. Although Otani et al.^3^ defined CME as hyporeflective spaces separated by hyperreflective septa, there is no quantitative value for the cyst diameters when defining CME in the hitherto literature.

Another definition used between clinicians is cystoid macular degeneration (CMD). CMD was first defined in chronic retinal vein occlusion, in a histopathological experimental study.^4^ CMD was seen to develop after CME with associated photoreceptor cell loss.^4^ CMD was also defined in chronic central serous chorioretinopathy (CSCR) as cystoid spaces in optical coherence tomography (OCT) with no fluorescein leakage.^5^ This term was always related with poorer visual acuity.^5,6^ In age-related macular degeneration (AMD), CMD was also defined as the presence of degenerated pseudocyst or retinal degeneration that had intraretinal cystic spaces.^7^ However, it is important to differentiate between CME and CMD in AMD because degenerative cysts are not an indicator of the activity of the lesion.

The aim of this study was to define and characterize CMD in DME, for which there is no clear definition. CMD is thought to be the result of chronic edema and may be associated with an unfavorable visual outcome. Determination of DME characteristics is necessary to predict visual prognosis and select appropriate treatment.

## Materials and Methods

In this retrospective cross-sectional study, an evaluation was made of 398 OCT scans of the eyes of 223 patients who were followed up with the diagnosis of DME at Gazi University Department of Ophthalmology between November 2011 and March 2015. A total of 259 eyes of 187 patients (92 female/95 male) who met the inclusion criteria were included in the study. The study was approved by the Local Ethics Committee of Gazi University.

Eyes with clinically significant macular edema as defined by ETDRS, that had fundus fluorescein angiography (FFA) and high-quality spectral OCT were included in the study. If the patient had received any prior treatment, OCT images obtained at least 3 months after the last treatment (intravitreal injection and/or laser therapy) were included in the study. Eyes with visually significant cataract or any other pathology causing visual deterioration such as corneal opacity, significant vitreous hemorrhage, optic atrophy, amblyopia, macular edema due to other causes such as uveitis, retinal vein occlusion and concurrent macular degeneration, or macular hole were excluded from the study. Eyes which had undergone cataract surgery within the last 6 months were also excluded from the study to exclude Irvine-Gass syndrome.

The demographic features of the patients (age, gender, diabetes duration) and stage and duration of DR were recorded. Best corrected visual acuity (BCVA) measured with Snellen chart, fundus examination, OCT, and FFA images were evaluated. BCVA was converted to LogMAR for statistical analysis. All OCT scans and FFA investigations were performed with Heidelberg Spectralis OCT (Spectralis; Heidelberg Engineering, Heidelberg, Germany).

Macular ischemia and type of edema were noted from FFA images. Macular ischemia was defined as an enlarged foveal avascular zone (≥1000 µm) or capillary non-perfusion areas within one disc diameter distance of the foveal center.^8^

Edema was classified as focal, diffuse, or mixed type. Focal edema was defined as local leakage areas with well-defined borders, and the term diffuse edema was used when large, poorly defined leakage areas affected the fovea. If there were features of both types of leakage, the edema was defined as mixed type.^9,10^

Central subfield thickness (CSFT), defined as the average thickness in the central 1000 µm-diameter circle of the ETDRS grid in OCT, was used for quantitative analysis of DME.^11^ In qualitative analysis of OCT scans, the presence of intraretinal cystic changes, serous foveal detachment (SFD), vitreomacular interface (VMI) abnormalities, outer retinal layer defects [inner segment/outer segment (IS/OS) band, external limiting membrane (ELM)], integrity of the foveal contour, and internal reflectivity of the cysts were evaluated. Cystic changes were defined as round or oval areas of low reflectivity separated by hyperreflective septa.^3^ SFD was defined as shallow elevation of the posterior surface of the retina appearing as optically clear, dome-shaped areas between the neurosensorial retina and RPE on OCT scans.^12,13^

VMI abnormalities were evaluated in six groups, with modifications made to the classification of the International Vitreomacular Traction Study group.^14^

1. Total perifoveal detachment: Posterior vitreous surface is completely detached from the macular region but not from the optic disc as seen in OCT

2. Vitreomacular adhesion (VMA):

a) Focal VMA (area of adhesion ≤1500 µm)

b) Broad VMA (area of adhesion >1500 µm)

3. Epiretinal membrane

4. Vitreomacular traction (VMT):

a) Focal VMT (area of traction ≤1500 µm)

b) Broad VMT (area of traction >1500 µm)

Outer retinal layer defect was defined as a loss of continuity of either the ELM or IS/OS band in the central 0.1 mm of the fovea. Shadowing behind the cysts and hard exudates were separated carefully.^15^

Integrity of the foveal contour was evaluated in three groups: 1) normal foveal depression; 2) normal foveal depression was disturbed but had not disappeared completely (mild distortion), 3) the foveal depression was completely flat or elevated (severe distortion).

Internal reflectivity of the cysts was classified as hyporeflective if similar to the vitreous, isoreflective if similar to the retinal layers, or heterogeneous (as defined in an earlier study).^16^

**Figure 1:**
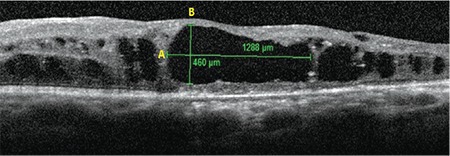
Example optical coherence tomography image showing measurements of the horizontal diameter A) and vertical diameter B) of the largest cyst

The largest cystoid space was determined for each eye and the horizontal and vertical diameters of the largest cyst in the area within 1000 µm of the foveal center were noted. All measurements were performed by the same investigator (N.G.Y.) using a manual caliper (Figure 1). To ensure high scan quality, scans with quality score <20 were not included in the evaluation. In addition, 25-line raster scans were carefully evaluated to make sure that there were no hyperreflective septa within our measurements of horizontal diameter of the largest cyst. Correlations between BCVA and the horizontal and vertical diameters of the largest cyst were analyzed using Pearson and Spearman correlation tests. The diameters were also analyzed for correlation with predetermined OCT and FFA findings.

The minimum BCVA to read newspaper print is known to be 20/60 (Snellen), and BCVA <20/60 is defined as severe visual loss.^17^ The relationship between BCVA and the horizontal and vertical diameters of the largest cyst resulting in severe visual loss was evaluated with receiver operating characteristic (ROC) analyses.

Cut-off values to differentiate the CME and CMD groups were determined from this analysis. The CME and CMD groups were created and the defined OCT and FFA findings were analyzed in the groups to determine the features of CMD.

### Statistical Analyses

Data obtained from the study were recorded using Excel for Windows (version 2010, Microsoft, Redmond, WA) and statistical analyses were performed using the Statistical Package for the Social Sciences for Windows (version 15.0, SPSS, Chicago, IL). The statistical level of significance was set to p<0.05. The Kolmogorov-Smirnov test, histograms, and P-P plots were used for the continuous variables to test the conformity to normal distribution. For comparisons of two groups, the independent samples t-test or Mann-Whitney U test were used according to the normality result. One-way ANOVA and LSD test for post-hoc analyses were used for comparisons of three or more groups if the variables were normally distributed, and the Kruskal-Wallis test was used if the variables were not normally distributed. The Mann-Whitney U test with Bonferroni correction was used for post-hoc analysis if the result revealed a significant difference. In correlation analysis, Pearson or Spearman correlation test were used according to the normality result. Pearson chi-square or Yate’s corrected chi-square tests were used for categorical variables. ROC curve analysis was used for the predictive value of the horizontal and vertical diameters of the largest cyst for severe visual loss.

## Results

A total of 259 eyes of 187 patients (49.2% women and 50.8% men) met the inclusion criteria. DME was detected in 138 right eyes (53.3%) and 121 left eyes (46.7%). There were 72 patients (38.5%) with bilateral involvement and 115 patients (61.5%) with unilateral DME. Other demographic features of the cases are shown in Table 1. There was history of prior intravitreal injection and/or laser therapy in 198 eyes (76.5%). The mean BCVA of the patients was 0.5±0.02 (0-1.6) LogMAR.

The mean CSFT was 474±131 (254-1121) µm. Cystic changes were detected in most cases (251 eyes, 96.9%). Therefore, the final analysis of the study was made in these 251 eyes. The horizontal diameter of the largest cyst did not conform to normal distribution while the vertical diameter of the largest cyst showed normal distribution. The median of the horizontal diameter was 433 (126-2213) µm and the mean of the vertical diameter was 305±142 (77-1059) µm.

**Figure 2:**
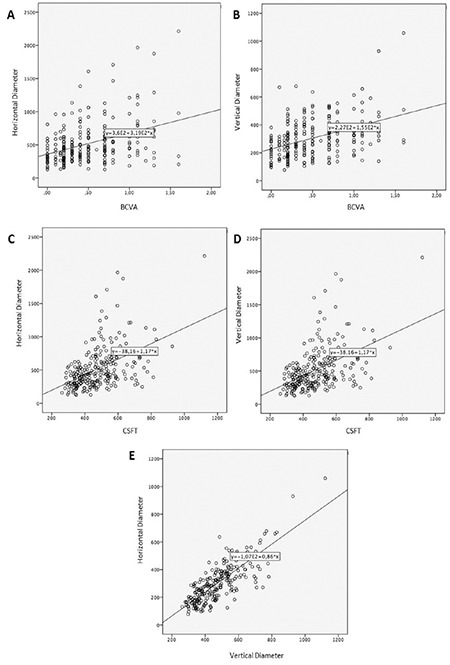
Correlation graphs for horizontal and vertical diameters of the largest cyst and best corrected visual acuity (BCVA) and central subfield thickness (CSFT): A) horizontal diameter and BCVA; B) vertical diameter and BCVA; C) horizontal diameter and CSFT; D) vertical diameter and CSFT; E) horizontal diameter and vertical diameter

The correlation between the horizontal and vertical diameters of the largest cyst and BCVA showed weak to moderate positive correlation (horizontal: r_s_=0.349; p<0.001, Figure 2a; vertical: r=0.419; p<0.001, Figure 2b). The correlation between the horizontal diameter of the largest cyst and CSFT showed moderate positive correlation (r_s_=0.487; p<0.001, Figure 2c). The correlation between the vertical diameter of the largest cyst and CSFT showed a medium to strong positive correlation (r_s_=0.798; p<0.001, Figure 2d). There was also a medium to strong positive correlation between the horizontal and vertical diameters (r_s_=0.678; p<0.001, Figure 2e).

Data in OCT and FFA related to the horizontal and vertical diameter of the largest cyst are given in Table 2. There was a statistically significant difference in the median horizontal diameter of the largest cyst between edema types in FFA (p=0.035). When the binary comparisons of groups were examined, the median horizontal diameter of the largest cyst in the mixed edema group (471 µm) was significantly higher than that in the focal edema group 392 µm (p=0.01). Although the median horizontal diameter of the largest cyst in diffuse edema (441 µm) was higher than that in focal edema, the difference was not statistically significant (p=0.04, Bonferroni-corrected).

There was no statistically significant difference between the horizontal and vertical diameters of the largest cyst within the groups in terms of VMI anomalies, presence of SFD, or internal reflectivity of the cysts.

**Figure 3:**
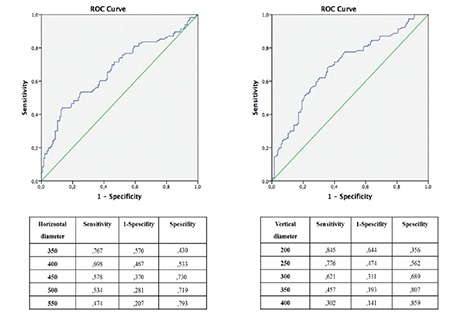
Receiver operating characteristic (ROC) curves and sensitivity/specificity values for the prediction of severe vision loss by A) horizontal diameter of the largest cyst (area under the curve=0.665; p<0.001) and B) vertical diameter of the largest cyst (area under the curve=0.692; p<0.001)

A cut-off value for the diameter of the largest cyst was needed to define CMD. To determine a cut-off value for the horizontal diameter of the largest cyst, a ROC curve was drawn, which has previously been reported to show that the horizontal diameter of the largest cyst can predict poor visual acuity. Assuming that other predictors were constant, the horizontal diameter of the largest cyst at 450 µm predicts visual acuity loss with 58% sensitivity and 73% specificity. The predictive ROC model showed moderate significance (AUC=0.665; 95% CI=0.597-0.733; p<0.001, Figure 3a).

The same analyses were applied using the vertical diameter of the largest cyst to determine another cut-off value to define CMD. A ROC curve was drawn, which has been reported to show that the vertical diameter of the largest cyst can predict poor visual acuity. Assuming that other predictors were constant, the vertical diameter of the largest cyst at 300 µm predicted visual acuity loss with 62% sensitivity and 69% specificity with moderately significant predictive power (AUC=0.692; 95% CI=0.626-0.757; p<0.001, Figure 3b).

Using these cut off-values, eyes with a horizontal diameter of the largest cyst ≥450 µm were classified as CMD (CMD-H group) and those with <450 µm were classified as CME (CME-H group), while eyes with vertical diameter of the largest cyst ≥300 µm and <300 µm were classified as CMD (CMD-V group) and CME (CME-V group), respectively.

OCT and FFA parameters related to these CME and CMD groups are shown in Table 3. In comparing these two groups, there were no statistically significant differences in terms of VMI anomalies, presence of SFD, or internal reflectivity of the cysts.

## Discussion

The definition of CMD was previously made for CSCR and AMD, but there has been no reported definition in DME. It is important to define CMD and identify the related features to have more information about the prognosis of edema and the benefit of treatment. It has been associated with poor visual acuity in CSCR, and is said to affect the need for treatment in AMD.^5,6,7^

Cyst formation begins with intercellular fluid accumulation. Coalescence of the extracellular fluid occurs due to the disruption of Müller cells, whose pump-like function keeps the macula dry.^18^ In the chronic stage, fluid accumulates intracellularly. The subsequent death of Müller cells and neuroglia results in the formation of large cystoid cavities.^19^ Otani et al.^3^ reported the acute and chronic morphologies of DME in an OCT-based study and stated that the disappearance of septa resulted in confluent large cysts which might fill all layers of the retina. Consistent with this pathogenesis, Yamomoto et al.^20^ found that eyes with CME had lower visual acuities than other types in OCT. In light of these data, it can be considered that in the chronic phase of cyst formation, the horizontal diameter of the cyst enlarges, damaging the adjacent retina. This data formed the basis of this study to define CMD. Also, Das et al.^18^ showed that eyes which had cystic changes with septa had higher visual acuity values than eyes which had nonseptated cystic spaces.

The baseline characteristics of our study, such as mean age and male predominance, were similar to the TURK-DEM study, which reflected the overall DME patients in Turkey.^21^ The results of that study showed a moderate degree of correlation between the horizontal diameter of the largest cyst and visual acuity (LogMAR). This was encouragement for further analysis. Similarly, a moderate degree of correlation was determined between the vertical diameter of the largest cyst and visual acuity (LogMAR) (correlation coefficients; r_s_=0.35, r=0.42, relatively). The high correlation of the two diameters of the cyst can be interpreted as enlargement of the cyst in these two planes. In a previous study, height of the foveal cystoid space and inferior subfield retinal thickness were found to be correlated.^22^ Retinal thickening in the inferior quadrant in particular was interpreted as a result of the disrupted retinal integrity with long-term cystic changes and the accumulation of fluid in the inferior zone due to gravity.^23^ Consistent with these findings and relationships, we believe that cystoid degeneration occurs due to increased cystic changes in both the horizontal and vertical planes in prolonged edema.

As expected, there was medium to high correlation between the vertical diameter of the largest cyst and CSFT (correlation coefficient r_s_=0.798). Also, a moderate correlation between the horizontal diameter of the largest cyst and CSFT was observed (correlation coefficient r_s_= 0.487). Large cysts tend to form in the foveal region, which has no inner retinal layers.^24,25^ At the center of the fovea, where there are no inner layers, the retina is more vulnerable to the development of large cysts and increases in mechanical stresses when the axial expansion of cysts is not sufficient to compensate for the increased volume.^26^ In light of this pathogenesis and the findings of the current study, we hypothesize that cyst enlargement continues in the horizontal plane after the vertical expansion exceeds the capacity of the retina. This process occurs with the degeneration of the retina.

In some previously published studies, it has been suggested that cysts have a damaging effect on bipolar and ganglion cells.^17,27,28^ A relatively new definition, disorganization of retinal inner layers (DRIL), refers to the disruption of the bipolar, horizontal, and amacrine cell synaptic field. Consequently, transmission between photoreceptors and ganglion cell complex is impaired.^29^ DRIL was found to be responsive to anti-vascular endothelial growth factor therapy.^30,31^ Yohannan et al.^32^ reported that the presence of a cyst is associated with decreased retinal sensitivity independent of increased retinal thickness and IS/OS junction disruption.

The presence of macular cystoid spaces has been found to be predictive of reduction in BCVA, and larger cystoid spaces were found to be more disruptive than small ones in a previous study by Sophie et al.^33^ In another study, Koleva-Georgieva and Sivkova^34^ showed a negative correlation between BCVA and cystoid DME groups (patients were grouped according to the horizontal diameter of cystoid spaces: <300 µm as mild, 300-600 µm as intermediate, and >600 µm as severe cystoid DME). In the hitherto literature there is no reported definition of CME according to cyst size with a specific cut-off value using OCT. In the current study, it was also aimed to find a cut-off value to distinguish CMD from CME. With this finding, the starting point of the degenerative effect of a cyst can be determined. In the current study, the horizontal and vertical diameters of the largest cyst were higher in cases with outer retinal damage, macular ischemia, and diffuse or mixed edema. These conditions are known to be associated with poor visual prognosis.^35,36,37,38,39,40,41,42,43,44,45,46^

In this study, CME and CMD groups were formed according to the horizontal and vertical diameters of the largest cyst. Horizontal cyst diameter ≥450 µm was defined as CMD. In this group, sensitivity was 58% for the prediction of visual acuity <20/60. In other words, eyes with horizontal cyst diameter ≥450 µm had a 58% probability of visual acuity <20/60. Horizontal cyst diameter <450 µm was defined as CME and had 73% specificity for the prediction of visual acuity ≥20/60. Therefore, eyes with horizontal cyst diameter <450 µm had 73% probability of visual acuity ≥20/60. Vertical cyst diameter ≥300 µm was defined as CMD. In eyes with vertical cyst diameter ≥300 µm, the probability of visual acuity <20/60 (sensitivity) was 62%. Vertical cyst diameter <300 µm was defined as CME, and the probability of visual acuity ≥20/60 (specificity) was 69% in this group.

A higher rate of outer retinal damage was observed in the CMD groups than in the CME groups. Likewise, macular ischemia was detected at a higher rate in the CMD groups than in the CME groups. Degeneration and macular ischemia seem to be related processes. In a previously published study, vascular hyperpermeability and ischemia were shown to cause necrosis and apoptosis in the neuroglia cells and this process resulted in large cystoid cavities. A vicious circle ensues with the enlargement of the cystoid spaces causing enlargement of the foveal avascular zone and increased foveal ischemia.^47^ In addition, the occurrence of cystoid spaces in the capillary nonperfusion areas and lack of reperfusion of these areas after the resolution of the chronic cystoid edema was shown in a previous OCT angiography study.^48^ The findings of the current study, which showed that more degeneration findings are present in ischemic cases, supported these data.

In the current study, the integrity of the foveal contour was also studied with an expectation that the presence of normal foveal contour or mild distortion were more likely to be associated with CME groups and severe distortion with the CMD groups. Indeed, our results showed that severe distortion of the foveal contour was more prevalent in CMD groups. Although Jun et al.^49^ evaluated foveal contour integrity in various ocular diseases, the current study is the first to use this parameter for grouping and analysis in DME to define CMD.

In a previous study, Liang et al.^16^ showed that accumulations in a cyst isoreflective to the plexiform layer were associated with fibrin and inflammatory debris. The heterogeneous reflectivity group of our study refers to this finding. Higher reflectivity of cystoid spaces was observed by Barthelmes et al.^50^ in exudative diseases such as DME compared to the normal vitreous and cystic spaces in degenerative diseases.

Although there was no supporting finding in our study, we hypothesize that the internal reflectivity of the cysts was related with chronicity and degeneration. Initially, the cyst is usually isoreflective. Then, cyst reflectivity becomes heterogeneous on OCT due to the debris accumulation as a result of degeneration. The degenerated cyst becomes hyporeflective in the chronic stage. In an earlier study supporting our hypothesis, the optical intensity of the cystoid space was thought to be a finding indicating chronicity.^51^ Contradictory to our hypothesis, the mean density of cystoid spaces was found to be negatively correlated with retinal sensitivity.^52^ Further studies are needed to clarify the relationship between the reflectivity of cystoid spaces and the degeneration process.

To the best of our knowledge, this is the first study to determine characteristics of CMD in DME. With these findings, degenerative cases can be diagnosed early in the management of DME. There were some limitations of this study. One is the retrospective design, and another is that there was no follow-up or evaluation of the treatment which was administered. The interpretation of some data such as foveal contour may contain bias. This subjective evaluation can be considered one of the limitations of the study. Future prospective studies investigating treatment response in eyes with CMD with follow-up information would be helpful to predict eyes with poor visual prognosis (such as those with CMD) and eyes with CME, which probably have better prognosis.

## Conclusion

In conclusion, cystoid degeneration in DME can be defined as the largest cyst in the central fovea having a horizontal diameter >450 µm and vertical diameter >300 µm, especially with concomitant macular ischemia, outer retinal damage, loss of foveal contour, and diffuse/mixed edema subtype. This definition can provide a new perspective to professionals in patient assessment and may help estimate the benefit of treatment.

## Figures and Tables

**Table 1:**
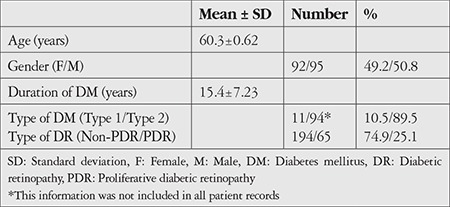
The demographic features of the patients

**Table 2:**
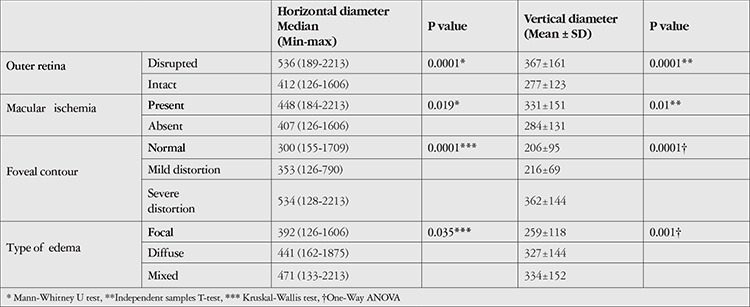
Optical coherence tomography and fundus fluorescein angiography parameters associated with the horizontal and vertical diameters of the largest cyst

**Table 3:**
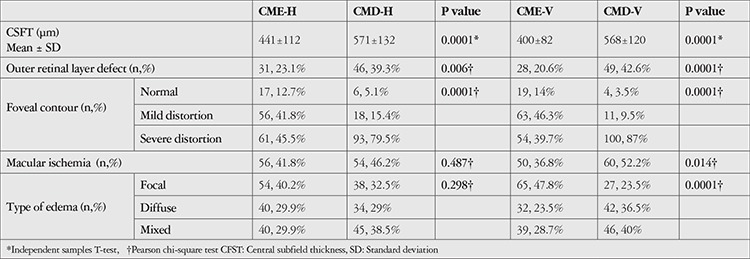
Optical coherence tomography and fundus fluorescein angiography parameters in cystoid macular edema (CME) and cystoid macular degeneration (CMD) groups based on horizontal (-H) and vertical (-V) diameters of the largest cyst
